# The need for a personalized approach for prostate cancer management

**DOI:** 10.1186/s12916-015-0344-1

**Published:** 2015-05-09

**Authors:** JP Michiel Sedelaar, Jack A Schalken

**Affiliations:** Radboud University Medical Center, Nijmegen, The Netherlands

**Keywords:** Biomarker, Genome, Individualized medicine, Prognosis, Prostate cancer

## Abstract

The stratification of patients for treatment of prostate cancer is based on very general parameters like prostate-specific antigen, Gleason score, and TNM classification. We use these rough parameters for selection of active surveillance, active treatment, and even for the treatment selection in metastasized or castration-resistant prostate cancers. Up to now, we have not used individualized genetic classifiers for detailed sub-stratification, thus treating all patients as equal, and being only moderately successful. With the expected increase in systemic treatments, there is an apparent need for such classifiers. We will address these needs in this short commentary.

## Background

In current urology practice, most men diagnosed with prostate cancer will present with local or locally advanced disease. They will be candidates for curative treatment with either radical surgery or radiation therapy. Despite this shift towards diagnosis at a more favorable clinical stage of prostate cancer, between 20% and 30% of these patients will have a biochemical relapse and need additional therapy [1]. At that point, most patients will first receive some form of salvage therapy, although a considerable number of patients will eventually need androgen deprivation therapy to treat metastasized prostate cancer. Androgen deprivation therapy is not a curative option in the treatment of prostate cancer, leading to castration-resistant prostate cancer and eventually death from prostate cancer in most cases.

The description of this clinical history is not uncommon, and underlines the limitations we encounter in determining the prognosis of individuals with prostate cancer. There is a clear, unmet need for reliable and reproducible prognostic biomarkers that can stratify patients on prognosis and identify who could benefit from early adjuvant treatment instead of salvage treatment.

### Current diagnostic and prognostic tools

The current diagnostic tools we mostly use are serum prostate-specific antigen (PSA), digital rectal exam, ultrasound in combination with guided biopsy, and histopathological Gleason grading. Of these diagnostic tools, only PSA can be classified as a biomarker and has limited performance status [2]. Because of these limitations in predicting prognosis, the treatment dilemma can lead to over-treatment, as well as under-treatment. Treating all patients in the adjuvant setting will lead to a considerable percentage of unnecessary complications and side effects (over-treatment), whereas treating patients only in a salvage setting will lead to relative late treatments and chance for failure of this treatment (under-treatment).

Research efforts have been undertaken to improve the prognostic stratification of the individual patient. Several markers complementary to PSA have been introduced and investigated. Most notable are the prostate health index, the OPKO 4K score, and PCA3 [3,4]. Although all of these biomarkers are promising in predicting biopsy outcome, none of these tests has been able to adequately predict the biological potential of the cancer in individual patients. Combining one or several of these biomarkers with state-of-the-art imaging modalities like multiparametric magnetic resonance imaging or PSMA-positron emission tomography combined with computed tomography could be considered as a step in the right direction.

Hence, there is an urgent need for reproducible prognostic biomarkers as an adjunct to histopathological grading. Gleason grading is still one of the more robust diagnostic tools and often used in individual clinical decision-making despite high intra- and inter-observer variability. Additionally, there have been significant changes in evaluation criteria, especially in the most frequent Gleason 3 and 4 patterns from which most treatment dilemmas (‘to treat or not to treat’) arise.

### Molecular prognostic tools

Genomic and molecular classifiers could greatly help with these previously mentioned clinical dilemmas. The best-evaluated example of such a molecular classifier is the Oncotype DX in the field of breast cancer. Oncotype DX (Genomic Health, Inc., Redwood City, CA, USA) is a tissue-based gene expression-profiling test that was first marketed in the US in 2004. It was designed to measure the 10-year risk of tumor recurrence in early-stage breast cancer at the time of initial diagnosis. Risk is reported as a 21-gene signature or recurrence score on a scale of 0 to 100. It is widely used in the US although the clinical benefits are still not completely understood [5].

For prostate cancer, two new molecular classifiers are now available for aiding decision-making for either active surveillance or intervention: Prolaris (cell cycle progression score) [6] and the Oncotype DX Genomic Prostate Score [7]. Both tests have been introduced for clinical decision-making for patients with low- or intermediate-risk prostate cancer who can choose between active surveillance or intervention. The tests are available as laboratory-developed tests by Clinical Laboratory Improvement Amendments laboratories. However, the treatment dilemma for patients with intermediate- or high-risk prostate cancer (that is, whether to give adjuvant (radio) therapy) has not yet been thoroughly addressed for the above-mentioned genomic test.

In their recently published article, Den *et al*. [8] describe the use of a validated 22-marker prostate cancer genomic classifier (Decipher) to identify patients with intermediate- or high-risk prostate cancer who could benefit from early adjuvant radiotherapy, compared to salvage treatment radiotherapy, after initial radical surgery. The 22-marker panel genomic classifier (GC) has been validated in previous studies and is performed on patient tissue specimens. For all patients, the Cancer of the Prostate Risk Assessment Post-Surgical (CAPRA-S) score was determined as was the Stephenson 5-year nomogram survival probability in addition to the GC score. In comparison with both the CAPRA-S and the Stephenson nomogram, the GC was the most prominent predictor of metastasis using a variety of statistical probability tests. A reclassification analysis showed that 71 patients (43%) with average- or high-risk CAPRA-S scores had their risk downgraded to GC low risk, and 69 of these patients (96%) remained metastasis-free on study follow-up. No information is given about possible biochemical recurrence in these patients. These findings illustrate the utility of an individualized and tailored approach in the management of intermediate- and high-risk post-prostatectomy patients with adverse histopathological findings.

There are some limitations in the presented study. In this retrospective study the decision for adjuvant radiotherapy was variable and differed among clinicians, while the decision for salvage radiotherapy was more guideline based. With this subjective selection it is impossible to conclude that the timing of early adjuvant treatment based on a genomic classifier is the main reason for the improved outcome. Also the use of androgen deprivation therapy adjuvant to the prostatectomy in some patients was non-guideline based. The ‘adjuvant’ use of hormonal treatment could have influenced the downgrading of the stage, serving as a stage migration, while not influencing the genomics. This could again explain the positive outcomes for the GC group receiving adjuvant treatment. In a prospective setting, with strict criteria for additional hormonal treatment and guidelines for when to proceed with adjuvant treatment, the conclusions would be more robust. Lastly, in this study there was no control group without radiotherapy adjuvant to radical prostatectomy. Without this control group no conclusions can be made on the benefit of the ‘additional’ radiotherapy compared to no radiotherapy.

## Conclusions

**Figure 1:**
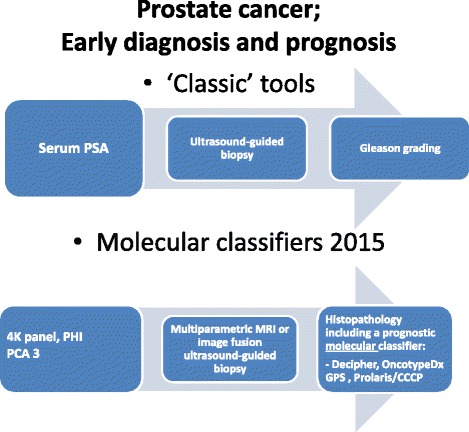
Schematic presentation of the classic steps in the early diagnosis of prostate cancer and the same approach implementing recent US Food and Drug Administration-approved tests or those offered by CLIA laboratories. CCCP, cell cycle control panel; GPS, Genomic Prostate Score; MRI, magnetic resonance imaging; PCA3, prostate cancer gene 3; PHI, Prostate Health Index; PSA, prostate-specific antigen.

Den *et al*. [8] presented an interesting new personalized approach for intermediate- and high-risk patients for whom adjuvant or salvage radiotherapy post-prostatectomy is indicated. Using a GC, these patients can be guided towards a personalized treatment approach, as illustrated in Figure 1. In the future, these findings must be confirmed in prospective trials. Another more compelling future study design could be the use of the GC-Decipher test as an add-on in systemic treatment studies for pre- and post-chemotherapy interventions. Identifying patients who can benefit the most from new systemic treatments could greatly improve the chances for survival for selected patients with castration-resistant prostate cancer.
